# Seroepidemiologic evidence of Q fever and associated factors among workers in veterinary service laboratory in South Korea

**DOI:** 10.1371/journal.pntd.0010054

**Published:** 2022-02-02

**Authors:** Dilaram Acharya, Ji-Hyuk Park, Jeong-Hoon Chun, Mi Yeon Kim, Seok-Ju Yoo, Antoine Lewin, Kwan Lee

**Affiliations:** 1 Department of Preventive Medicine, College of Medicine, Dongguk University, Gyeongju, Korea; 2 School of Public Health, University of Montreal, Montreal, Canada; 3 Medical Affairs and Innovation, Héma-Québec, Montreal, Quebec, Canada; 4 Division of Bacterial Disease, Bureau of Infectious Disease Diagnosis Control, Korea Disease Control and Prevention Agency, Cheongju, Korea; 5 Faculty of Medicine and Health Science, Sherbrooke University, Sherbrooke, Quebec, Canada; Instituto de Pesquisas Veterinarias Desiderio Finamor, BRAZIL

## Abstract

The incidence of Q fever has rapidly increased in South Korea since 2015. This study was undertaken to investigate the seroprevalence and seroreactivity of Q fever and the risk factors associated with its seroprevalence among workers in the veterinary service laboratory (VSL) in South Korea. This seroepidemiologic study was conducted in a total of 661 human subjects out of 1,328 subjects working in 50 VSL existing in South Korea between July 15 and July 29, 2019. Data were collected by administering survey questionnaires and by analyzing collected blood samples to determine the presence of antibodies against *Coxiella burnetii*. The seroprevalence and seroreactivity of *C*. *burnetii* infection were determined based on serum titers as (phase II IgG ≥1:256 and/or IgM ≥1:16) and (phase II IgG ≥1:16 and/or IgM ≥1:16) as determined by indirect immunofluorescent assay. Work, work environment, behavioral risk and protective factors associated with seroprevalence of Q fever were assessed by employing multivariable logistic regression analysis. Among the 661, the seroprevalence and seroreactivity of *C*. *burnetii* infection were 7.9% and 16.0%, respectively. Multivariate logistic regression analysis showed the risk factors significantly associated with seroprevalence were the antemortem inspection of cattle, goats, or sheep (APR (adjusted prevalence ratio), 2.52; 95% CI, 1.23–4.70)), animal blood splashed into or around eyes (APR, 2.24; 95% CI, 1.04–4.41), and contact with animals having Q fever (APR, 6.58; 95% CI, 3.39–10.85) during the previous year. This study suggests the need for precautions when contact with cattle, goats, or sheep is expected, especially during the antemortem inspection, when dealing with *C*. *burnetii* infected animals, or when there is a risk of ocular contact with animal derivatives. Therefore, we recommend the consistent use of appropriate personal protective equipment and other protective measures including PPE treatment and washing of body surfaces after work to prevent *C*. *burnetii* infections among VSL staff in South Korea.

## Author summary

This study investigated the seroprevalence and seroreactivity of Q fever and determined the risk factors associated with seroprevalence of Q fever among the nationally representative sample of workers in the veterinary service laboratory (VSL) in South Korea. We found the seroprevalence and seroreactivity of *C*. *burnetii* infection among VSL staff were 7.9% and 16.0%, respectively. In addition, the work-specific tasks such as antemortem inspection of cattle, goats, or sheep, contact with animals having Q fever, and having animal blood splashed into or around eyes were found to be significantly associated with the seroprevalence of Q fever. Our findings suggest precautions be taken when contacting cattle, goats, or sheep, especially during the antemortem inspection, and dealing with *C*. *burnetii* infected animals, and when there is a risk of ocular contact with animal derivatives, such as the use of appropriate personal protective equipment, and compliance with other measures that prevent *C*. *burnetii* infections among VSL staff in South Korea.

## Introduction

Q fever (also known as Query fever) is an acquired infectious disease caused by the obligate intracellular bacterium *Coxiella burnetii*, which reportedly has a worldwide distribution with the exceptions of Antarctica and New Zealand [1–3]. Cattle, goats, and sheep are the usual primary reservoirs of *C*. *burnetii*, and infections can easily be disseminated to human being from infected animals [4,5]. Furthermore, a growing number of animals including domestic mammals, marine mammals, reptiles, ticks, and birds have been reported to shed the bacterium [3]. Shedding often occurs from birth products, urine, feces, and the milk of infected animals [1,6–8]. *C*. *burnetii* can survive even under harsh environmental conditions such as in extremely hot and cold climates and in dry locations for months to years [9,10].

The usual modes of transmission are via direct or indirect exposure to aerosolized materials from infected animals or birth products or due to the ingestion of unpasteurized milk [11–14]. The major risk groups are those that contact animals regularly, such as livestock farmers, slaughterhouse workers, veterinarians, meat processing workers, and laboratory workers [15–20]. However, *C*. *burnetii* can travel several kilometers in the wind and can cause Q fever in people not in direct contact with infected animals or their body fluids [2,9,10].

Animals infected with *C*. *burnetii* are usually asymptomatic, but in others, the bacteria can cause problems such as miscarriage, stillbirth, low birth weight, and weak offspring [2,21]. In human, Q fever is usually asymptomatic; clinical features include influenza-like illness, though deaths have been reported. However, life-threatening complications like severe pneumonia and hepatitis can be accompanied by severe neurological abnormalities (meningitis) or heart-related illnesses (e.g., myocarditis, pericarditis, thrombosis, and hemophagocytic syndrome) [1,2,22]. Although antibiotics such as doxycycline and hydroxychloroquine have been to be effective in individuals with uncomplicated *C*. *burnetii* infection [23], no licensed vaccine is available for preventing *C*. *burnetii* infection, except in Australia. In Netherlands, a limited vaccination campaign was conducted in 2011 for people at risk of developing endocarditis and heart valve infection, but of those, only 11% and 18%, respectively, were vaccinated [24,25].

In South Korea, the first confirmed case of Q fever was reported in 1992 [26], and in 2006, Q fever was designated a notifiable infectious disease. Over the period 2006 to 2015, its average annual incidence was around 10 cases/year, but after 2015, the number of Q fever cases dramatically increased to reach 163 cases in 2018 [27]. Given that animal husbandry is of the major occupation in South Korea [28] and the distribution of *C*. *burnetii* in animals has been reported to be widespread (seroprevalence in Korean cattle 9.5–11.6% and in goats 15–19%) [29–32], *C*. *burnetii* infection represents a potential long-standing problem that cannot be neglected.

Further, various seroprevalence results for Q fever in nationally representative samples of different risk groups have been reported in some earlier South Korean studies. For example, a seroprevalence of 1.0% of 511 veterinarians in 45 veterinary service laboratories in 2011 [33], and a seroreactivity of 11.0% (134/1,222) was reported in dairy cattle farmers [34] and of 9.1% (84/923) to 11.3% (151/1503) among slaughterhouse workers [15,35]. We considered that determination of the seroprevalence of Q fever and the identification of its associated risk factors among populations at risk would have policy implications for the prevention and control of *C*. *burnetii* infection among human subjects. Due to gaps in knowledge and a lack of recent data, we undertook this study to investigate the seroprevalence and seroreactivity of Q fever and risk factors associated with seroprevalence among workers in the veterinary service laboratory (VSL) in South Korea.

## Methods

### Ethics statement

The study protocol was approved by the Institutional Review Board of Dongguk University Gyeongju Hospital (approval number:110757-201906-HR-02-03). Investigators provided a full description of the study, including its aims and objectives, procedures, risk, and benefits to all participants before study commencement. Written informed consent was obtained from all participants, who were informed they could decide to participate or not in the study, to respond or not to questionnaire items, and to decide whether to submit a blood sample without prejudice. All personal identifiers were removed before data analysis.

### Study design, subjects, and sampling

This seroepidemiologic study was conducted on a nationally represented sample of an at-risk population for zoonotic diseases, that is, on VSL workers in South Korea. According to the Korean Ministry for Food, Agriculture, Forestry, and Fisheries, there were a total of 50 VSLs in South Korea. In these 50 VSL, 1,328 workers worked in different departments who came into close contact with livestock in 2019. The distribution of VSL workers by the type of VSLs (S1 Table), and work activities details of VSLs have been enclosed as supplementary information (S1 File). All workers in VSL in South Korea were considered as eligible study participants irrespective of gender or job description and had no contraindication of drawing blood sample unless having health conditions such as infected or scarred tissues, and those who consented to participate in the study willingly and voluntarily. To obtain a representative sample, we estimated the required sample size using a reported seroprevalence of 1% for *C*. *burnetii* infection in a previous Korean nationwide seroepidemiologic survey conducted on VSL workers in 2011 [33]. The sample size was determined employing formula for the cross-sectional study [36,37],

$$n0=\frac{z2pq}{e2}*1.0*\frac{99.0}{(3)2}=380$$

where n0 = the unadjusted sample size required, z = 1.96 = standard normal variate at 95% level of confidence, p = 1%: proportion of seroprevalence for *C*. *burnetii* infection among VSL workers, q = 1-p: proportion of seroprevalence negative for *C*. *burnetii* infection VSL workers, and e = acceptable error willing to be committed giving the clinical importance level of 1%. Thus, the unadjusted sample size was n0 = 380. Because the nationwide population of VSL workers was known (N = 1,328), applying finite population, corrected formula for proportions, the minimum sample needed was estimated using formula: n = no/1+[(no-1)/N] = 296. This number was then adjusted by taking into consideration an anticipated cluster sample design effect of 2. Therefore, adjusted sample size = n* design effect = 296*2 = 592. This estimate was increased to 651 by adding 10% to cover exclusions, absenteeism, and dropouts. Finally, we enrolled a total of 661 VSL workers in the study.

These sampled subjects (661 VSL workers) were selected out of 1,328 VSL workers from the 50 VSL using probability proportional to size (PPS) sampling method. The required number of subjects to be selected from each VSL offices was estimated by calculating sampling fraction (n/N). Then, the subjects were approached convincingly till required number of samples were obtained. If the subjects refused to participate, then next subject within the cluster were approached and selected. Furthermore, if estimated number of subjects were not reached from the same VSL due to refusal or they were absent at the time of survey, subjects were enrolled from subsequent VSL (S1 Table).

### Data collection

During the development of the questionnaires, researchers examined in detail the job descriptions and job specifications of the study participants to develop, define, and re-define the test instrument. The questionnaires were formulated by adapting the questionnaires used in a previous study conducted in 2011 on VSL staff[33] and using information from other studies[15,35,38,39]. The questionnaires included questions that addressed subject general characteristics, work and work environmental issues, the use of personal protective measures during work, and other presumed risk factors related to exposure to *C*. *burnetii*. (S1 Questionnaire). Data were collected using a pre-tested and structured questionnaire between July 15, 2019, and July 29, 2019. Questions regarding the questionnaire items were answered by a trained interviewer during on-site visits.

### Exposure assessment

From July 15 to 29, 2019, four teams visited the 50 VSL. Each team consisted of five members, that is, two blood sample collectors, a questionnaire surveyor, a general manager, and a supervisor. Each team made on-site visits to each VSL at a mutually convenient time and answered participant questions. Exposures to risk factors were determined by asking whether participants had been exposed to work-related environmental factors, e.g., drawing blood samples, burying and killing, general disinfection, postmortem examination/autopsies, pathological appraisal/morbidity examinations, serum test, antemortem inspection of livestock (cattle, goats, sheep, pigs, chickens), dismantling inspection of slaughter, microbiological examination, residual material examination, raw milk examination, mastitis examination, livestock products prospection, egg test, administrative work, frequency of contact with animals during past one year, work-situation, and exposure to animal birth and abortion, and the use of personal protective equipment/instruments. Assessment of exposure was not limited to particular animals but assessed in general. Exposures to risk factors and the use or non-use of protective measures were dichotomized (yes vs. no or always vs. sometimes/rarely).

### Serologic testing

Blood samples (10 ml) were drawn on the same days the questionnaire was completed and placed in serum-separating tubes (SSTs). Serum was immediately separated by centrifugation, and samples were numbered, stored in sealed iceboxes containing icepacks, and transferred to the Korea Center for Disease Control and Prevention (KCDC) for serologic tests. A cold chain was maintained at 4°C for all blood and serum samples collected. The seroprevalence of Q fever was determined phase II IgG ≥1:256 and/or IgM ≥1:16, and seroreactivity was defined as phase II IgG ≥1:16 and/or IgM ≥1:16 as per existing practices recommended by KCDC [40], by using indirect immunofluorescence assay (IFA) (Focus Diagnostics, Cyprus, CA, USA).

### Statistical analysis

Statistical analysis was carried out using SPSS version 22.0 (SPSS, IBM, Armonk, NY, USA). Univariate logistic regression analysis was conducted to assess the association between potential risk/protective factors and seroprevalence of Q fever among VSL staff.

In multivariate logistic regression model, we entered for only those variables that were considered/presumed to be at risk of having *C*. *burnetii* infection with significance level <0.05. The model was fitted using backward elimination method. The Wald test was used to determine statistical significance for each of the independent variables. The goodness of fit of the final logistic regression model was observed by using Hosmer-Lemeshow statistic and was found to be satisfactory (p>0.05). Adjusted and unadjusted prevalence ratio with 95% confidence intervals were presented. A p-value <0.05 was considered statistically significant.

## Results

### Serologic test results

**Fig 1:**
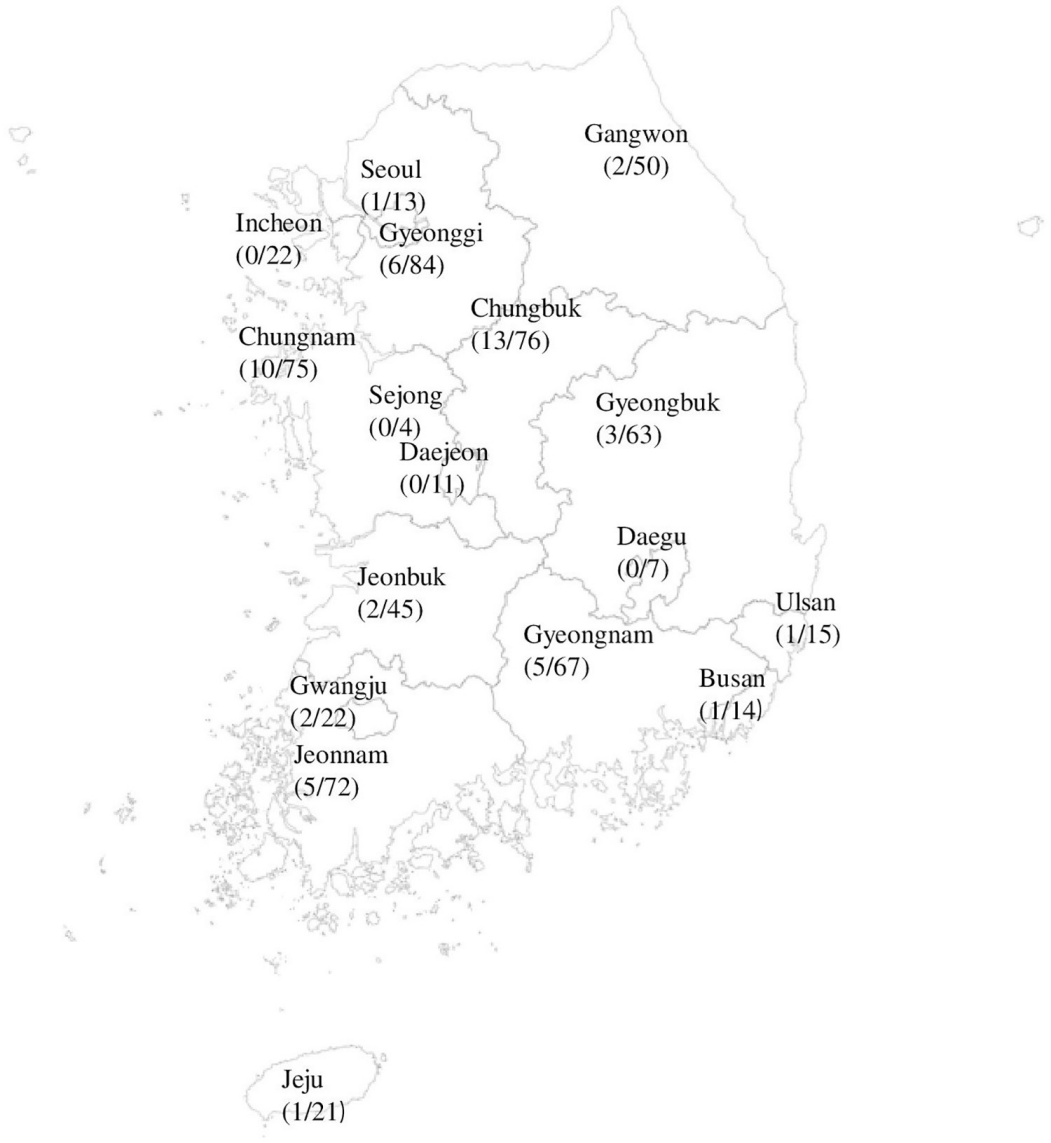
Distribution of Q fever Seroprevalence cases/ selected total numbers of VSL workers from existing VSL in selected locations, south Korea (The map was created with statistical geographic information system (SGIS) which is free location-based open service platform (https://sgis.kostat.go.kr/statexp/view/index#) accessed on October 20, 2021. The seroprevalence cases out of total number of VSL workers on map was created using, Microsoft Excel 2019).

Table 1 shows the seroprevalence and seroreactivity of Q fever among the 661 participants. Out of 661 study participants, 52 (7.9%) had seroprevalence of Q fever (IgG ≥1:256 and/or IgM ≥1:16) and 106 (16.0%) were seroreactive (IgG ≥1:16 and/or IgM ≥1:16) test results performed by IFA method. Of total 52 Q fever seroprevalent subjects, majority of them were from the VSL offices situated in Chungbuk (13), Chungnam (10), and Gyeonggi (6) region of South Korea (Fig 1).

**Table 1:** Seroreactivity and seroprevalence of Q fever among veterinarians working in veterinary service laboratory in South Korea, 2019.

Variables	Serum titer	N = 661 (%)
**Seroreactivity**		
Reactive	(IgG≥1:16 and or IgM ≥1:16)	106 (16.0)
Non-reactive	(IgG<1:16 and or IgM<1:16)	555 (84.0)
**Seroprevalence**		
Positive	(IgG≥1:256 and or IgM≥1:16)	52 (7.9)
Negative	(IgG<1:16 and or IgM<1:16)	609 (92.1)

N; numbers, Ig; immunoglobin

### Personal characteristics of the study subjects associated with Q fever seroprevalence by univariate analysis

Participants’ personal characteristics such as the male gender (prevalence ratio (PR), 2.12; 95% CI, 1.10–4.33), those who were working as veterinarians (PR, 2.30; 95% CI, 1.10–5.38), and current smokers (PR, 2.02; 95% CI, 0.98–3.87) had significantly higher rates of seroprevalence of Q fever. However, those who not aware about Q fever and human brucellosis (PR, 0.40; 95% CI, 0.12–1.00), and those subjects who were not educated about Q fever and human brucellosis (PR, 0.52; 95% CI, 0.26–0.99) were found to have significantly lower rates of Q fever seroprevalence than their counterparts. Other important characteristics such as those of subject age, years spent working in a VSL, type of VSL, region, and education level were not found to be significantly associated with the seroprevalence of Q fever (Table 2).

**Table 2:** Personal profiles of the study participants associated with Q fever seroprevalence among workers in veterinary service laboratory in South Korea, 2019.

Variables	Total	Seroprevalence, N (%)	Prevalence Ratio (95%CI)	*p-value* *
**Gender**				
Male	387	39 (10.1)	2.12 (1.10–4.33)	0.016
Female	274	13 (4.7)	Reference	
**Age in years**				
21–29	180	15 (8.3)	Reference	
30–39	153	14 (9.2)	1.09 (0.49–2.53)	0.801
≥40	328	23 (7.0)	0.84 (0.42–1.73)	0.602
**Duration of work in years**				
< 5	395	27 (6.8)	Reference	
5-<15	149	14 (9.4)	1.37 (0.66–2.71)	0. 332
≥15	117	11 (9.4)	1.37 (0.61–2.86)	0. 379
**Type of Veterinary Service Laboratory (VSL)**				
Main VSL	266	21 (7.9)	1.70 (0.62–5.78)	0.277
Branch VSL and other VSL	287	26 (9.1)	1.95(0.73–6.52)	0.161
Institute of Health and Environment VSL	108	5 (4.6)		
**Region**				
Northern	169	9 (5.3)	Reference	
Central	281	28 (10.0)	1.87 (0.85–4.50)	0.096
Southern	211	15 (7.1)	1.33 (0.54–3.45)	
**Veterinarians**				
Yes	445	43 (9.7)	2.30 (1.10–5.38)	0.018
No	215	9 (4.2)	Reference	
**Nationality**				
Korean	660	52 (7.9)	Reference	
Foreigner	1	0 (0.0)	NA	
**Marital status**				
Married	260	22 (8.5)	Reference	
Others (single/ divorced/ bereavement)	401	30 (7.5)	0.88 (0.49–1.60)	0.660
**Educational level**				
Elementary/middle/high school	80	3 (3.8)	Reference	
University	455	43 (9.5)	2.52 (0.80–12.69)	0.108
Graduate school	125	6 (4.8)	1.29 (0.31–5.33)	0.726
**Visited overseas within past one year**				
Yes	311	24 (7.7)	0.96 (0.53–1.72)	0. 897
No	350	28 (8.0)	Reference	
**Smoking**				
Current smokers	95	14 (14.7)	2.02 (0.98–3.87)	0.020
Others (none/past smokers)	561	38 (6.8)	Reference	
**Alcohol consumption**				
Alcohol consumption (current)	358	26 (7.3)	0.83 (0.45–1.52	0.524
Others (none/past)	264	23 (8.7)	Reference	
**(Awareness) heard of Q Fever**				
No	138	5 (3.6)	0.40 (0.12–1.00)	0. 045
Yes	522	47 (9.0)	Reference	
**(Awareness) heard of brucellosis**				
No	26	1 (3.8)	0.47 (0.01–2.78)	0.454
Yes	634	51 (8.0)	Reference	
**Educated about Q Fever and human brucellosis**				
No	272	14 (5.1)	0.52 (0.26–0.99)	0. 037
Yes	389	38 (9.8)	Reference	

* Using univariate logistic regression, N, number; CI confidence interval, NA, not applicable.

### Univariate analysis of specific work-related factors associated with Q fever seroprevalence among workers in VSL

Results showed that specific work-related factors, that is, those who performed blood drawing for all types of animals (PR, 2.31; 95% CI, 1.20–4.72) and types of animals (cattle, goats, or sheep) (PR, 2.16; 95% CI, 1.18–4.07) were significantly associated with a higher seroprevalence of Q fever than those who were not in such works. Furthermore, those who were in the pathological appraisal work of all types of animals (PR, 1.73; 95% CI, 0.96–3.10) was found to have a significantly higher seroprevalence rates of Q fever than those who were not in such works. Significantly higher seroprevalence of Q fever were also found among participants involved in the antemortem inspection of all livestock (PR, 1.97; 95% CI, 1.04–3.58) and the antemortem inspection of cattle, goats, or sheep (PR, 2.22; 95% CI, 1.16–4.07). Likewise, higher rates of seroprevalence of Q fever with statistically significant difference were also found to those study participants who were involved in dismantling inspection of slaughter of all animals (PR, 2.04; 95% CI, 1.08–3.72), and dismantling inspection of cattle, goats, or sheep (PR, 2.26; 95% CI, 1.19–4.16) (Table 3).

**Table 3:** Univariate analysis of specific work-related factors associated with seroprevalence of Q fever among workers in veterinary service laboratory in South Korea, 2019.

Variables	Total	Seroprevalence, N (%)	*PR*	*p-value* *
**Livestock protection-related factors**				
**Drawing blood samples**				
Yes	372	39 (10.5)	2.31 (1.20–4.72)	0.007
No	287	13 (4.5)	Reference	
**Drawing blood sample (cattle, goats, or sheep)**				
Yes	307	34 (11.1)	2.16 (1.18–4.07)	0.006
No	352	18 (5.1)	Reference	
**Burying and killing**				
Yes	103	13 (12.6)	1.78 (0.87–3.41)	0.066
No	552	39 (7.1)	Reference	
**Burying and killing of cattle, goats, or sheep**				
Yes	89	11 (12.4)	1.72 (0.79–3.40)	0.105
No	571	41 (7.2)	Reference	
**General disinfection**				
Yes	217	22 (10.1)	1.48 (0.81–2.65)	0.157
No	439	30 (6.8)	Reference	
**General disinfection of cattle, goats, or sheep**				
Yes	172	18 (10.5)	1.50 (0.79–2.74)	0. 163
No	473	33 (7.0)	Reference	
**Postmortem examination(autopsy)**				
Yes	269	49 (18.2)	1.24 (0.83–1.85)	0.263
No	389	57 (14.7)	Reference	
**Postmortem examination(autopsy) of cattle, goats, or sheep**				
Yes	194	20 (10.3)	1.49 (0.81–2.69)	0. 155
No	464	32 (6.1)	Reference	
**Pathological appraisal**				
Yes	240	26 (10.8)	1.73 (0.96–3.10)	0.044
No	416	26 (6.3)	Reference	
**Pathological appraisal of cattle, goats, or sheep**				
Yes	187	21 (11.2)	1.70 (0.93–3.06)	0.055
No	471	31 (6.6)	Reference	
**Serum test**				
Yes	414	38 (9.2)	1.60 (0.85–3.20)	0.126
No	245	14 (5.7)	Reference	
**Serum test of cattle, goats, or sheep**				
Yes	337	30 (8.9)	1.29 (0.72–2.34)	0.361
No	319	22 (6.9)	Reference	
**Inspection of slaughter-related factors**				
**Antemortem inspection of all livestock**				
Yes	140	18 (12.9)	1.97 (1.04–3.58)	0.017
No	521	34 (6.5)	Reference	
**Antemortem Inspection of cattle, goats, or sheep**				
Yes	118	17 (14.4)	2.22 (1.16–4.07)	0. 005
No	540	35 (6.5)	Reference	
**Dismantling inspection of slaughter of all animals**				
Yes	136	18 (13.2)	2.04 (1.08–3.72)	0.012
No	525	34 (6.5)	Reference	
**Dismantling inspection of slaughter of cattle, goats, or sheep**				
Yes	116	17 (14.7)	2.26 (1.19–4.16)	0. 004
No	542	35 (6.5)	Reference	
**Microbiological examination**				
Yes	205	15 (7.3)	0.90 (0.45–1.68)	0.735
No	456	37 (8.1)	Reference	
**Microbiological examination of cattle, goats, or sheep**				
Yes	165	14 (8.5)	1.06 (0.53–2.01)	0.829
No	479	38 (7.9)	Reference	
**Residual material examination**				
Yes	166	14 (8.4)	1.09 (0.54–2.07)	0.763
No	495	38 (7.7)	Reference	
**Residual material examination of cattle, goats, or sheep**				
Yes	137	12 (8.8)	1.11 (0.53–2.17)	0.737
No	510	40 (7.8)	Reference	
**Raw milk examination**				
Yes	61	6 (9.8)	1.28 (0.44–3.01)	0. 564
No	600	46 (7.7)	Reference	
**Mastitis examination**				
Yes	74	5 (6.8)	0.84 (0.26–2.11)	0. 717
No	587	47 (8.0)	Reference	
**Inspection of processed livestock products**				
Yes	78	3 (3.8)	0.45 (0.09–1.41)	0.177
No	583	49 (8.4)	Reference	
**Egg test**				
Yes	116	7 (6.0)	0.73 (0.27–1.63)	0.438
No	545	45 (8.3)	Reference	
**Administrative work**				
Yes	346	30 (8.7)	1.24 (0.69–2.25)	0.440
No	315	22 (7.0)	Reference	
**Other work**				
Yes	19	2 (10.5)	1.35 (0.15–5.14)	0.674
No	642	50 (7.8)	Reference	

* Using univariate logistic regression, N, number; CI confidence interval.

Table 4 presents the association between exposure to work-situation risk factors within past one year and seroprevalence of Q fever by univariate analysis among workers in VSL. Exposure to a number of work-situation-related factors demonstrated positive association of risk of having seroprevalence of Q fever among study participants with higher prevalence ratio such as animal blood splashed into or around eyes (PR, 2.19; 95% CI, 1.03–4.34), animal blood splashed around body (PR, 1.94; 95% CI, 0.97–4.20), contact with animal faeces/urine around the body (PR, 2.58; 95% CI, 1.24–6.03), contact with animals having Q fever (PR, 9.17; 95% CI, 4.62–17.79), tested samples (blood, organelles, or tissues) with animals having Q fever (PR, 5.33; 95% CI, 2.77–9.98), and contact with animal with brucellosis (PR, 1.96; 95% CI, 1.01–3.65).

**Table 4:** Associations between work-situation-related exposure factors during past one year and seroprevalence of Q fever among workers in veterinary service laboratory in South Korea, 2019.

Variables	Total	Seroprevalence, N (%)	PR (95% CI)	*p-*value*
**Animal blood splashed into or around eyes**				
Yes	85	12 (14.1)	2.19 (1.03–4.34)	0.016
No	528	34 (6.4)	Reference	
**Animal blood splashed around mouth**				
Yes	87	9 (10.3)	1.48 (0.62–3.14)	0.285
No	517	36 (7.0)	Reference	
**Animal blood splashed around body**				
Yes	419	40 (9.5)	1.94 (0.97–4.20)	0.046
No	224	11 (4.9)	Reference	
**Contact with animal faeces/urine around eyes**				
Yes	166	16 (9.6)	1.41(0.72–2.66)	0.257
No	455	31 (6.8)	Reference	
**Contact with animal faeces/urine around mouth**				
Yes	145	16 (11.0)	1.78 (0.90–3.38)	0.060
No	468	29 (6.2)	Reference	
**Contact with animal faeces/urine around the body**				
Yes	419	43 (10.3)	2.58 (1.24–6.03)	0.007
No	227	9 (3.7)	Reference	
**Presence of any injury on skin**				
Yes	356	35 (9.8)	1.57(0.86–3.00)	0.119
No	273	17 (6.2)	Reference	
**Needle injury**				
Yes	315	32 (10.2)	1.71(0.95–3.16)	0.054
No	338	20 (5.9)	Reference	
**Contact with animals having Q fever**				
Yes	38	17 (44.7)	9.17 (4.62–17.79)	<0.0001
No	492	24 (4.9)	Reference	
**Tested samples (blood, organelles, or tissues) with Q fever**				
Yes	61	18 (29.5)	5.33(2.77–9.98)	<0.0001
No	506	28 (5.5)	Reference	
**Availability of microbiological work bench**				
Yes	577	45 (7.8)	0.80 (0.25–4.05)	0.716
No	31	3 (9.7)	Reference	
**Contacted with animal with brucellosis**				
Yes	113	16 (14.2)	1.96 (1.01–3.65)	0.023
No	472	34 (7.2)	Reference	
**Tested samples with brucellosis**				
Yes	259	28 (10.8)	1.67 (0.92–3.04)	0.064
No	356	23 (6.5)	Reference	
**Involved in animal birth**				
Yes	27	2 (7.4)	0.940.11–3.57)	0.930
No	634	50 (7.9)	Reference	
**Involved in the management of animal abortion**				
Yes	123	14 (11.4)	1.61(0.80–3.04)	0.123
No	538	38 (7.1)	Reference	

* Using univariate logistic regression, N, number, CI confidence interval.

Table 5 shows the association between use of personal protective measures within past one year and seroprevalence of Q fever by univariate analysis among workers in VSL. However, use of personal protective measures (always vs. rarely/sometimes) was not found to be significantly associated with the seroprevalence of Q fever.

**Table 5:** Univariate analysis of associations between use of personal protective measures during past one year and seroprevalence of Q fever among workers in veterinary service laboratory in South Korea, 2019.

Variables	Total	Seroprevalence, N (%)	PR (95% CI)	*p-*value***
**Wearing protective glasses**				
Always	36	5 (13.9)	1.84 (0.57–4.60)	0.187
Others*	623	47 (7.5)	Reference	
**Wearing protective mask**				
Always	213	19 (8.9)	1.20 (0.64–2.18)	0. 510
Others*	447	33 (7.4)	Reference	
**Types of protective health mask (KF94 grade or higher)**				
Always	31	2 (6.5)	0.83 (0.09–3.19)	0.803
Others**	609	47 (7.7)	Reference	
**Wearing protective gloves**				
Always	539	43 (8.0)	1.07 (0.51–2.50)	0. 848
Others*	121	9 (7.4)	Reference	
**Wearing a protective apron**				
Always	106	7 (6.6)	0.81 (0.30–1.81)	0.606
Others*	553	45 (8.1)	Reference	
**Wearing protective boots**				
Always	321	30 (9.3)	1.43 (0.79–2.60)	0.198
Others*	337	22 (6.5)	Reference	
**Wearing protective clothing**				
Always	415	35 (8.4)	1.21 (0.66–2.31)	0.508
Others*	245	17 (6.9)	Reference	
**Disinfected after-work instruments**				
Always	445	32 (7.2)	0.84 (0.46–1.60)	0.572
Others*	212	18 (8.5)	Reference	
**Maintained after-work personal hygiene (disinfection or bathing)**				
Always	374	31 (8.3)	1.11 (0.62–2.04)	0.695
Others*	283	21 (7.4)	Reference	

*** Using univariate logistic regression

*sometimes/rarely

**surgical, cloth dust mask or no use of mask, N, number; CI; confidence interval.

### Multivariable analysis of specific work-related factors associated with Q fever seroprevalence among workers in VSL

Table 6 shows the multivariable logistic regression analysis for factors associated with Q fever seroprevalence among workers in VSL in South Korea, 2019. All significant variables in univariate analysis (p <0.05) that were related to usual animal contact, work, and work-related factors and those not exhibiting multicollinearity were entered in adjusted final multivariable logistic regression model with stepwise backward elimination. Study participants involved in antemortem inspection of cattle, goats, or sheep during past one year were found to have significantly higher risk of being seroprevalence at univariate analysis, and this association remained significant in the adjusted model (adjusted prevalence ratio (APR), 2.52; 95% CI, 1.23–4.70). Animal blood splashed into or around eyes during past one year was also found to be significant risk factor in the final adjusted model (APR, 2.24; 95% CI, 1.04–4.41). Likewise, contact with animals having Q fever was also significantly predicted the seroprevalence of Q fever (APR, 6.58; 95% CI, 3.39–10.85).

**Table 6:** Multivariable logistic regression analysis of factors associated with Q fever seroprevalence among workers in veterinary service laboratory in South Korea, 2019.

Variables	Total, N	Seroprevalence, N (%)	APR (95%CI)	*p-*value
**Antemortem inspection of cattle, goats, or sheep during past one year**				
Yes	118	17 (14.4)	2.52 (1.23–4.70)	0. 011
No	540	35 (6.5)	Reference	
**Animal blood splashed into or around eyes during past one year**				
Yes	85	12 (14.1)	2.24 (1.04–4.41)	0. 037
No	528	34 (6.4)	Reference	
**Contact with animals having Q fever during past one year**				
Yes	38	17 (44.7)	6.58 (3.39–10.85)	< 0. 001
No	492	24 (4.9)	Reference	

N, number; APR, adjusted prevalence ratio; CI, confidence interval. Significant variables in univariate analysis (p<0.05) included in final multivariable logistic regression model were: gender, veterinarians, smoking, awareness about Q fever, educated about Q fever and brucellosis, drawing blood sample (cattle, goats, or sheep), antemortem inspection of cattle, goats, or sheep, dismantling inspection of slaughter of cattle, goats, or sheep, animal blood splashed into or around eyes, animal blood splashed around body, contacted with animal faeces/urine around the body, contact with animals having Q fever, tested samples (blood, organelles, or tissues) with Q fever, contacted with animal with brucellosis.

## Discussion

On many occasions, *C*. *burnetii* infection goes unnoticed because the majority are asymptomatic or are misdiagnosed as some other febrile illnesses [41–43]. Therefore, knowledge of the extent of covert infectious diseases and their risk factors in high-risk groups is a prerequisite for the development of targeted health interventions. In the present study, the seroprevalence and seroreactivity of *C*. *burnetii* infection among the 661 VSL workers that participated were 7.9% and 16.0%, respectively. In addition, multivariable logistic regression analysis demonstrated that the regular antemortem inspection of cattle, goats, or sheep during the previous year, work-situation-related factors such as experience of animal blood being splashed into or around eyes and contact with Q fever infected animals during the previous year were significantly associated with the seroprevalence of *C*. *burnetii* infection.

Serology is a commonly used, relatively simple means of detecting acute and chronic human *C*. *burnetii* infections in seroepidemiologic studies [24], and IFA is considered as reference laboratory method for diagnosing *C*. *burnetii* infection in man, especially for seroprevalence studies [1,44]. Our findings regarding the seroprevalence (7.9%) and seroreactivity (16.0%) of *C*. *burnetii* infection are comparable with those of previous Korean studies on nationally representative high-risk groups that reported seroprevalence of 10.2% for slaughterhouse workers [15], 11.0% for dairy cattle farmers [34], and 9.1% for cattle slaughterhouse workers [35]; in all of these studies, *C*. *burnetii* seroreactivity was defined as a phase II antigen IgG or IgM titer of ≥1:16. However, one South Korean study conducted in 2011 reported a seroprevalence of Q fever of only 1.0% among VSL staff [33], which is considerably less than that found in the current study. Despite having used same criteria used to detect the *C*. *burnetii* infection as recommended by KCDC in South Korea, *C*. *burnetii* infection rates have been on rise since 2015. Given that animal husbandry is one of the major occupations in recent years in South Korea [28], and the literatures support the fact that increasing status of common zoonoses such as Q fever in animals can result into such an incremental infection among human subjects as well who are at risk by occupation. Although we could not have exact information about to what extent of animals infected with *C*. *burnetii* at the moment, we assume that incremental *C*. *burnetii* infection since 2015 in South Korea might be as a result of increased *C*. *burnetii* infection factories and farms, and the growing interest in Q fever among infectious disease physicians.

Nevertheless, all South Korean studies conducted on various at-risk subjects have suggested that *C*. *burnetii* infection is endemic among high-risk groups and advocated for specific interventions to reduce the endemicity of *C*. *burnetii* infection among such high-risk groups. Inevitably, occupationally exposed individuals are more vulnerable to *C*. *burnetii* infection than members of the general population. A Japanese study reported diverse seroprevalence for Q fever among the general population and high-risk groups like veterinarians and meat-processing workers (3% in healthy adults vs. 11–22% in high-risk occupations) [45]. Likewise, another recent study from Estonia [46] reported significantly higher seroprevalence of *C*. *burnetii* antibodies in veterinary professionals (9.62%) and dairy cattle farmers (7.73%) than in the general population (3.9%). In South Korea, almost all seroepidemiologic study have been conducted among at risk population. To the best of researchers’ knowledge, there are no such studies being performed in general population at national level. The only one peer-reviewed paper published in 2006 reported that an overall seroprevalence (antibody titers IgG ≥1:256) of 1.5% (3/205) among asymptomatic people screened in a rural area of South Korea. These results demonstrate a continuous need to monitor staff at risk to institute surveillance programs and to initiate interventions at the earliest stage.

Reported seroprevalence of *C*. *burnetii* infection among at-risk populations in other countries vary considerably, for example, 16% in Egypt [47] among subjects living in agricultural areas, 26% among blood donors in Namibia [48], 12% in farmers and abattoir workers in Turkey [49], 16% among abattoir workers in Australia [50], and 47.2% among women occupationally exposed to livestock in Denmark [51]. Thus, it appears the seroprevalence of *C*. *burnetii* infection among South Korean VSL workers is lower than in other countries. We attribute these variable seroprevalence to geographies, regions, prevalence of animal infections, types and extents of exposure to reservoirs of infection, and discrepancies between serological assessment techniques and the cut-off values used.

Work settings, job types, and the working environments of individuals that deal with animals and animal products influence exposure statuses and the statuses of zoonotic infections. For example, an Australian study showed working on the disposal of deceased cats or dogs and participating in the euthanasia of cats or dogs were associated with the greatest risk of contracting Q fever [52], while another study concluded working on goat farms presented the greatest risk [53]. We examined several work and work environment-related risk and protective factors associated with the seroprevalence of Q fever, and found specific work-related factors, namely, the routine antemortem inspection of cattle, goats, or sheep during the previous year were associated with a higher prevalence ratio of Q fever infection, which we suggest was due to the airborne transmission of *C*. *burnetii* from infected live animals during inspections to determine the medical fitness of animals for slaughter. In addition, participants that routinely contacted animals with Q fever during the previous year were also at significantly higher risk of *C*. *burnetii* infection than their counterparts. However, we could not determine the modes of transmission of *C*. *burnetii*. A similar study conducted in the USA among veterinarians routinely involved in the treatment of cattle, swine, or wildlife also reported a higher seroprevalence of *C*. *burnetii* infection [54]. Work-related activities such as milking cattle, providing general healthcare and birth assistance to cattle, and contact with still-born animals were also found to be significant risk factors of the presence of antibodies against *C*. *burnetii* in a study conducted in cattle farmers and farm residents in three northeastern Mongolian provinces [55]. Several other studies support the notion that the exposure to ruminant animals and their products can predict seropositive test results of Q fever among occupationally exposed persons such as veterinarians, abattoir workers and slaughterhouse workers, and animal husbandry workers [15,17,19,20,46].

Interestingly, the present study showed that being splashed by animal blood in or around eyes during the previous year was positively associated with the seroprevalence of Q fever. Previous Korean studies performed on high-risk occupational groups have reported similar findings [15,34]. In a previous Korean study conducted by Chu et al. [15] experience of having cattle blood splashed around the mouth within the previous two weeks increased the risk of seropositivity for Q fever, while in the current study, animal blood splashed into/around eyes during the previous year was associated with significantly higher risk. In another Korean study [34] on dairy farmers, ocular exposure to birth products during calf delivery was found to be a risk factor of *C*. *burnetii* seroreactivity. It has been well established that large numbers of *C*. *burnetii* are excreted in urine, vaginal fluids, feces, and milk, and that the bacterium is present at high concentrations in the birth fluids and placentas of infected cattle and small ruminants [1,56–59]. Importantly, *C*. *burnetii* can be isolated from the blood, lungs, liver, spleen, mammary gland of domestic animals, based on the chronicity of infection [60]. It can be presumed that blood splashed from infected domestic animals to VSL workers in our study might contribute to pose incremental risk of Q fever seroprevalence.

Our univariate analysis demonstrated that VSL workers educated/heard about Q fever appeared to be at risk. However, this association could not remain in final adjusted model. Additionally, always using protective measures including wearing a protective mask, were not found to have significantly associated with lower risk of Q fever seroprevalence in this study although protective masks (N-95 or above) have been known as a protective factor for *C*. *burnetii* infection [3,61]. We assume that VSL workers in South Korea might insufficiently use appropriate protective masks and other protective measures. However, since the most important way to prevent zoonotic diseases is to use of protective equipment, there is need for PPE treatment and washing of body surfaces after work.

The present study has a number of limitations that warrant consideration. First, we did not determine the *C*. *burnetii* infection statuses of animals, and environmental screening of animal farms and thus, we could not link animal infection rates and human infection statuses. Second, we could not investigate associations between exposure to animal-specific body fluids and *C*. *burnetii* infections, although we did identify several exposure factors. Further integrative studies between veterinary and VSL workers are required to understand modes of infection and to support the adoption of appropriate control measures. Third, the three significant variables in the multivariate logistic regression analysis that are linked to outcome variable have occurred within a year. It has been well documented that there could be variation in detectable antibody responses of Q fever infection by the period of presence of antibodies in seropositive people. It has been reported that the median time of onset of IgG phase II antibody response was 5 (0–182) days with half time median 937 days, while median time to onset for IgM phase II antibody response was 14 (0–205) days with half time median 400 days. Thus, this could have impacted on our estimates of outcome of interest [62,63]. Further, follow-up serological and clinical studies in this concern should therefore be recommended. Fourth, we collected blood samples from July 15, 2019 to July 29, 2019, and seasonality may have influenced study outcomes [9,10]. Therefore, we recommend further periodic studies be conducted considering the influence of seasonality.

Despite these limitations, we believe the results of this study are meaningful because they identify relationships between *C*. *burnetii* infection status and specific risk factors in a high-risk, nationally representative population, which means our findings can be generalized in South Korea and similar settings and have policy implications. The specific targeted interventions such as vaccination to animal, and human risk groups, and periodic health appraisal should therefore be considered with more emphasis on possible risk exposure status.

Summarizing, this study shows the seroprevalence and seroreactivity of *C*. *burnetii* infection measured by IFA among VSL workers were 7.9% and 16.0%, respectively. The risk factors found to be significantly associated with the seroprevalence of Q fever were: the antemortem inspection of cattle, goats, or sheep, having animal blood splashed into or around eyes, and contact with animals having Q fever during the previous year. In order to reduce the *C*. *burnetii* infection rate among VSL workers, precautions should be made during contact with animals, especially during the antemortem inspection, and additional precautions should be taken to protect staff from ocular contact with animal derivatives when they are expected to contact cattle, goats, or sheep suspected to have Q fever. In addition, there is need for PPE treatment and washing of body surfaces after work. Furthermore, comparative seroepidemiologic studies should be performed to determine the natures of relations between the infection statuses of animal, environmental, and humans and the prevalence of *C*. *burnetii* infection in high-risk groups with a view toward making robust policy recommendations.

## Supporting information

S1 Table Distribution of Veterinary Service Laboratory Offices by type of VSLs, locations, and sample enrolled in the study. (DOCX)

S1 File Job Description of VSL workers. (DOCX)

S1 Questionnaire Seroepidemiologic study of Q fever among veterinary service laboratory workers in South Korea, 2019. (DOCX)
