# Supplementary material for: Seroepidemiologic evidence of Q fever and associated factors among workers in veterinary service laboratory in South Korea
Source: PLoS Negl Trop Dis. 2022 Feb 2;16(2):e0010054. doi: 10.1371/journal.pntd.0010054 (PMC8809587; doi:10.1371/journal.pntd.0010054)
Supplement: S1 Table — (DOCX) [file pntd.0010054.s001.docx]

S1 Table: Distribution of Veterinary Service Laboratory Offices by type of VSLs, locations, and sample enrolled in the study

| **Area** | **Type of VSL and existing VSL workers** | | | | **Total**  **VSL workers** | **Sample to be selected**  **by PPS** | **Sample enrolled** | **Positive among sampled subjects** |
| --- | --- | --- | --- | --- | --- | --- | --- | --- |
|  | Veterinary Service Laboratory Head offices | Branch Veterinary Service Laboratory | Other Veterinary Service Laboratory Organization | Institute of Health and Environment Veterinary Service Laboratory |  |  |  |  |
| Seoul | - | - | - | 23 | 23 | 11 | 13 | 1 |
| Busan | - | - | - | 19 | 19 | 9 | 14 | 1 |
| Daegu | - | - | - | 28 | 28 | 14 | 7 | 0 |
| Incheon | - | - | - | 38 | 38 | 19 | 22 | 0 |
| Gwangju | - | - | - | 25 | 25 | 12 | 22 | 2 |
| Daejeon | - | - | - | 23 | 23 | 11 | 11 | 0 |
| Ulsan | - | - | - | 25 | 25 | 12 | 15 | 1 |
| Sejong | - | - | - | 16 | 16 | 8 | 4 | 0 |
| Gyeonggi | 97 | 63 | 10 | - | 170 | 85 | 84 | 6 |
| Gangwon | 41 | 59 | - | - | 100 | 50 | 50 | 2 |
| Chungbuk | 63 | 41 | 11 | - | 115 | 57 | 76 | 13 |
| Chungnam | 58 | 90 | - | - | 148 | 74 | 75 | 10 |
| Jeonbuk | 50 | 88 | 15 | - | 153 | 76 | 45 | 2 |
| Jeonnam | 53 | 53 | - | - | 106 | 53 | 72 | 5 |
| Gyeongbuk | 69 | 87 | - | - | 156 | 78 | 63 | 3 |
| Gyeongnam | 39 | 70 | 20 | - | 129 | 64 | 67 | 5 |
| Jeju | 54 | - | - | - | 54 | 27 | 21 | 1 |
| Total | 524 | 551 | 56 | 197 | 1,328 | 661 | 661 | 52 |
