# Supplementary material for: Seroepidemiologic evidence of Q fever and associated factors among workers in veterinary service laboratory in South Korea
Source: PLoS Negl Trop Dis. 2022 Feb 2;16(2):e0010054. doi: 10.1371/journal.pntd.0010054 (PMC8809587; doi:10.1371/journal.pntd.0010054)
Supplement: S1 File — (DOCX) [file pntd.0010054.s002.docx]

**S1 File: Job Description of VSL workers**

**a) Livestock protection:**

1. Drawing blood sample: animal blood collection (cattle, pig, chicken/duck, goat and sheep) is carried out in veterinary medicine for diagnostic and other purposes.

2. Burying and killing: this is done for all suspected meat or by-products or dead body of animals

3. General disinfection: disinfecting agents are registered by the Environmental Protection Agency (EPA) as “antimicrobial pesticides” and are substances used in the containment of harmful microorganisms (i.e., bacteria, viruses, or fungi) on inanimate objects and surfaces.

4. Postmortem examination/dead body examination (autopsy): this involved the examination of postmortem or dead body of animals

5. Pathological appraisal/morbidity examination: quickly and accurately analyzing the cause of livestock diseases, identifying the causes.

6. Serum test: blood serum is tested to all suspected samples to identify whether there is infection.

**b) Livestock inspection:**

1. Inspection of slaughter:

Identifying the slaughterhouses and individuals, and fully observe the general health, walking and standing status, and nutritional status of the livestock before slaughtering.

2. Dismantling inspection of slaughter: thoracic organs, abdominal organs and carcasses are examined during the slaughter of animals that have passed the Inspection of slaughter. all samples should be taken and further inspected in the laboratory to prevent distribution as raw material livestock products.

3. Microbiological examination: to provide hygienic raw material livestock products to consumers by conducting general bacteria and E. coli tests on carcasses slaughtered in the workplace

4. Residual material examination: animal medicine administered for the purpose of prevention and treatment of livestock diseases is slaughtered before being completely excreted after being absorbed into the body of the livestock. This is a test to promote food safety.

5. Raw milk examination: by inspecting the milk produced by the milking farmers by the state-certified institution, it ensures fairness in the payment of ties and implements the mastitis control business to promote safe and clean milk production and development of the dairy industry through oil quality improvement.

6. Mastitis examination: providing safe livestock products to consumers by preventing the damage of dairy farmers and preventing misuse of antibiotics by early detection of mastitis through early detection of mastitis-infected cows and selection of effective antibiotics

7. Livestock products prospection: upon receipt of products produced by the livestock products processing office, the sensory test, physicochemical test, microbial test, residual material test, and other tests are strictly conducted to inform whether the processing standards and ingredient specifications of livestock products prescribed by the Livestock Sanitation Control Act are met. Sampling products are randomly sampled at livestock processing establishments, meat packaging processing establishments, and distributors, and the product specifications, pathogenic microbiological tests, and residual material inspections are conducted for each product, and administrative measures (product recovery, business suspension, etc.). This is to secure food safety.

8. Egg test: it is a test for contributing to the safe supply of livestock food by investigating microorganisms and residues on eggs of livestock animals, i.e., chicken, ducks, and quail eggs.

9. Administrative work: those who perform the simple administrative work at offices.

10. Others: the other works other than above-mentioned.
