# Supplementary material for: Seroepidemiologic evidence of Q fever and associated factors among workers in veterinary service laboratory in South Korea
Source: PLoS Negl Trop Dis. 2022 Feb 2;16(2):e0010054. doi: 10.1371/journal.pntd.0010054 (PMC8809587; doi:10.1371/journal.pntd.0010054)
Supplement: S1 Questionnaire — (DOCX) [file pntd.0010054.s003.docx]

**S1 Questionnaire**

**General information**

Investigator/Surveyor (s):

Survey Date:

**Participants General information**

| Name: |  |
| --- | --- |
| Date of Birth/Age: | YY/MM/DD |
| Gender: | ◯ Male ◯ Female ◯ Other |
| Residence Address: |  |
| Home Phone #: |  |
| Cell Phone #: |  |
| Affiliation: Veterinary Service Laboratory | Department/Branch office, or Name of team |

♠ Check box

| Check blood sample collection | Confirm Questionnaires | Confirm receipt of gift certificate | Remarks |
| --- | --- | --- | --- |
|  |  |  |  |

**Work and work Environment-related information:**

| 1 | When did you start working as veterinarians in veterinary service laboratory? | **Year………** |
| --- | --- | --- |
| 1.1 | When did you work at your current job? | **Year/Month…...** |
| 2 | Which of the following tasks is your primary responsibility? | ① Preservation  ② Inspection  ③ Administration  ④ Other (specify)……… |

3. Please indicate whether you have performed each task for the past year and the animals you have been working on (multiple responses possible).

(☞ Select the animal only you have been working with)

| Name of the Job | | Response | | Working animals (multiple response allowed) | | | | |
| --- | --- | --- | --- | --- | --- | --- | --- | --- |
|  |  | Yes | No | Cow | Pig | Chicken/duck) | Goat | Sheep |
| **1. Livestock preservation** | | | | | | | | |
| 1.1 | Drawing blood samples | ① | ② | ◯ | ◯ | ◯ | ◯ | ◯ |
| 1.2 | Burying and killing | ① | ② | ◯ | ◯ | ◯ | ◯ | ◯ |
| 1.3 | General disinfection | ① | ② | ◯ | ◯ | ◯ | ◯ | ◯ |
| 1.4 | Cadaver examination (autopsy) | ① | ② | ◯ | ◯ | ◯ | ◯ | ◯ |
| 1.5 | Pathological appraisal | ① | ② | ◯ | ◯ | ◯ | ◯ | ◯ |
| 1.6 | Serum test | ① | ② | ◯ | ◯ | ◯ | ◯ | ◯ |
| **2. Livestock product inspection** | | | | | | | | |
| 2.1 | Inspection of slaughter | ① | ② | ◯ | ◯ | ◯ | ◯ | ◯ |
| 2.2 | Dismantling inspection (slaughter) | ① | ② | ◯ | ◯ | ◯ | ◯ | ◯ |
| 2.3 | Microbiological examination | ① | ② | ◯ | ◯ | ◯ | ◯ | ◯ |
| 2.4 | Residual material examination | ① | ② | ◯ | ◯ | ◯ | ◯ | ◯ |
| 2.5 | Raw milk examination | ① | ② | - | | | | |
| 2.6 | Mastitis examination | ① | ② | - | | | | |
| 2.7 | Inspection of processed livestock products | ① | ② | - | | | | |
| 2.8 | 2.8. Egg test | ① | ② | - | | | | |
| 3. Administrative work | | ① | ② | - | | | | |
| 4. others specify…………… | | ① | ② | - | | | | |

4. Please select the frequency of contact with animal(s) when you work for the past one year.

| Item | | Most  often | Often | Sometimes | Almost none | Not at all  none |
| --- | --- | --- | --- | --- | --- | --- |
| 4.1 | Cattle | ① | ② | ③ | ④ | ⑤ |
| 4.2 | Pig | ① | ② | ③ | ④ | ⑤ |
| 4.3 | Chicken (duck) | ① | ② | ③ | ④ | ⑤ |
| 4.4 | Goat | ① | ② | ③ | ④ | ⑤ |
| 4.5 | Sheep | ① | ② | ③ | ④ | ⑤ |
| 4.6 | Others specify (……………) | ① | ② | ③ | ④ | ⑤ |

5. These are the questionnaires about wearing personal protective equipment/measures within past one year (including laboratory). Please select the appropriate frequency for each question.

| Items | | Most  often | Often | Sometimes | Almost none | Not at all |
| --- | --- | --- | --- | --- | --- | --- |
| 5.1 | Do you wear protective glasses during work? | ① | ② | ③ | ④ | ⑤ |
| 5.2 | Do you wear a protective mask while working? | ① | ② | ③ | ④ | ⑤ |
| 5.3 | Do you wear protective gloves at work? | ① | ② | ③ | ④ | ⑤ |
| 5.4 | Do you wear protective aprons at work? | ① | ② | ③ | ④ | ⑤ |
| 5.5 | Do you wear protective boots at work? | ① | ② | ③ | ④ | ⑤ |
| 5.6 | Do you wear protective clothing at work? | ① | ② | ③ | ④ | ⑤ |
| 5.7 | Do all the instruments once used are thoroughly disinfected before the next use? | ① | ② | ③ | ④ | ⑤ |
| 5.8 | Do you maintain personal hygiene like disinfection, bathing, etc. after work? | ① | ② | ③ | ④ | ⑤ |

| 6. | What type of protective mask do you use at work (including laboratory) within past one year? | ① Surgical mask (disposable)  ② Cloth mask  ③Dustproof mask  ④ Health mask (KF80 grade)  ⑤ Health mask (KF94 grade or higher)  ⑥ Do not use any protective mask |
| --- | --- | --- |
| 7. | Is there a microbiological workbench (such as a biosafety or clean bench) in the laboratory? | ① Yes  ② No  ③ Do not know |

8. In the questions given below area about the work-situation within past one year. Please select the appropriate item.

| Items | | Yes | No | Do not know | |
| --- | --- | --- | --- | --- | --- |
| 8.1 | Have animal blood splashed into/around your eyes? | ① | ② | ③ | |
| 8.2 | Have animal blood splashed around your mouth? | ① | ② | ③ | |
| 8.3 | Have animal blood splashed around your body? | ① | ② | ③ | |
| 8.4 | Have animal faeces/urine been contacted with around your eyes? | ① | ② | ③ | |
| 8.5 | Have animal faeces/urine been contacted with around your mouth? | ① | ② | ③ | |
| 8.6 | Have animal faeces/urine been contacted with around the body? | ① | ② | ③ | |
| 8.7 | Have you ever had any injuries to your hands or skin? | ① | ② | ③ | |
| 8.8 | Have you ever been stabbed by the pointed weapons or the like during work? | ① | ② | ③ | |
| 8.9 | Have you ever been in contact with a Q fever infected animal? | ① | ② | ③ | |
| 8.10 | Have you ever tested the sample of (blood, organelles, tissue etc.) with Q fever? | ① | ② | ③ | |
| 8.11 | Have you ever contacted an animal with brucellosis? | ① | ② | ③ | |
| 8.12 | Have you ever tested samples (blood, etc.) with brucellosis? | ① | ② | ③ | |
| \| 9. Have you ever been involved in the birth of animals during the past one year? \| \| ① No, go to question # 10  ② Yes, answer question # 9.1 \| \| --- \| --- \| --- \| \| 9.1 \| What kind of animal was it? (Multiple responses possible) \| ◯ Cow  ◯ Pig  ◯ Goat  ◯ Sheep  ◯ Others specify……… \| \| 10. Have you ever been involved in treatment of animal abortion during the past one year? \| \| ① No, go to question # 11  ② Yes, answer question # 10.1 \| \| 10.1 \| What kind of animal was it? (Multiple responses possible \| ◯ Cow  ◯ Pig  ◯ Goat  ◯ Sheep  ◯ Others specify……… \| | | | | |  |

**Awareness-related information:**

| 11 | Have you ever heard of Q Fever? | ① No, go to question number 28  ② Yes, Go to question number 27 |
| --- | --- | --- |
| 12 | Please least all the methods/media from where you have learned/aware about Q fever | ◯ Newspapers  ◯ Broadcast (TV, radio)  ◯ Internet  ◯ Health promotion brochure  ◯ Work colleagues  ◯ Patients around  ◯ Public Health Center  ◯ Doctors or medical staff  ◯ Training (safety education, etc.) |
| 13 | Have you ever heard of brucellosis? | ① No, go to question number 30  ② Yes, Go to question number 29 |
| 14 | Please least all the methods/media from where you have learned/aware about human brucellosis | ◯ Newspapers  ◯ Broadcast (TV, radio)  ◯ Internet  ◯ Health promotion brochure  ◯ Work colleagues  ◯ Patients around  ◯ Public Health Center  ◯ Doctors or medical staff  ◯ Training (safety education, etc.) |
